# KnetMiner: a comprehensive approach for supporting evidence‐based gene discovery and complex trait analysis across species

**DOI:** 10.1111/pbi.13583

**Published:** 2021-04-05

**Authors:** Keywan Hassani‐Pak, Ajit Singh, Marco Brandizi, Joseph Hearnshaw, Jeremy D. Parsons, Sandeep Amberkar, Andrew L. Phillips, John H. Doonan, Chris Rawlings

**Affiliations:** ^1^ Rothamsted Research Harpenden UK; ^2^ IBERS Aberystwyth University Aberystwyth UK

**Keywords:** gene discovery, gene network, knowledge graph, knowledge discovery, exploratory data mining, data integration, candidate gene prioritization, information visualization, systems biology, bioinformatics

## Abstract

The generation of new ideas and scientific hypotheses is often the result of extensive literature and database searches, but, with the growing wealth of public and private knowledge, the process of searching diverse and interconnected data to generate new insights into genes, gene networks, traits and diseases is becoming both more complex and more time‐consuming. To guide this technically challenging data integration task and to make gene discovery and hypotheses generation easier for researchers, we have developed a comprehensive software package called KnetMiner which is open‐source and containerized for easy use. KnetMiner is an integrated, intelligent, interactive gene and gene network discovery platform that supports scientists explore and understand the biological stories of complex traits and diseases across species. It features fast algorithms for generating rich interactive gene networks and prioritizing candidate genes based on knowledge mining approaches. KnetMiner is used in many plant science institutions and has been adopted by several plant breeding organizations to accelerate gene discovery. The software is generic and customizable and can therefore be readily applied to new species and data types; for example, it has been applied to pest insects and fungal pathogens; and most recently repurposed to support COVID‐19 research. Here, we give an overview of the main approaches behind KnetMiner and we report plant‐centric case studies for identifying genes, gene networks and trait relationships in *Triticum aestivum* (bread wheat), as well as, an evidence‐based approach to rank candidate genes under a large *Arabidopsis thaliana* QTL. KnetMiner is available at: https://knetminer.org.

## Introduction

Genomics is undergoing a revolution. Unprecedented amounts of data are being generated to gain deeper insight into the complex nature of many traits and diseases (Boyle *et al.,* 2017; Stephens *et al.,* 2015). Paradoxically, the vast growing landscape of diverse and interconnected data can often hinder scientists from translating complex and sometimes contradictory information into biological understanding and discoveries. Searching for relevant information amongst larger and more complex data can take longer and so risks information being overlooked or subjective biases being introduced. Even after the gathered information is complete, it can be demanding to assemble a coherent view of how each piece of evidence might come together to ‘thread a story’ about the biology that can explain how genes and gene networks might be implicated in a complex trait or disease. New tools are needed to provide scientists with a more fine‐grained and connected view of the scientific literature and databases, rather than the conventional information retrieval tools currently at their disposal.

Scientists are not alone facing these challenges: knowledge searches form a core part of the duties of many professions. Studies have highlighted the necessity but significant challenge for search systems to give feedback and generate confidence, explainability and accountability (Russell‐Rose *et al.,* 2018). Search duration also influences human choice about whether to continue the task (Sweis *et al.,* 2018). When implemented well, search systems can accelerate research by cutting both the time and the cost of reviewing genes, traits and molecules of interest before initiating expensive experiments. Additionally, search systems offer a framework for the prioritization of future research, which can highlight gaps in knowledge.

Knowledge graphs (KG) are increasingly used to make search and information discovery more efficient (Fensel *et al.,* 2020). KGs are contributing to various Artificial Intelligence (AI) applications including link prediction, node classification, and both recommendation and question answering systems (Ali *et al.,* n.d.; Sheth *et al.,* 2019). KGs model heterogeneous knowledge domains by integrating information into advanced unified data schemas (i.e. ontologies) and leverage that to apply formal and statistical inference methods to derive new knowledge (Ehrlinger and Wöß, 2016). Compared to more traditional data models, knowledge graphs are very flexible at integrating and searching connected heterogeneous data, where data schemas are not established a‐priori (Yoon *et al.,* 2017), and often subject to frequent changes. KGs in various forms have been widely adopted in many disciplines, ranging from social sciences to engineering, physics, computer science, design and manufacturing. Many research laboratories, like us, are building biological KGs aimed at supporting crop improvement (Hassani‐Pak *et al.,* 2016; Xiaoxue *et al.,* 2019), drug target discovery (Mohamed *et al.,* 2019), disease gene prioritization (Alshahrani and Hoehndorf, 2018; Messina *et al.,* 2018) and COVID‐19 research (Reese *et al.,* 2021).

The integrated, semi‐structured and machine readable nature of KGs provides an ideal basis for the development of sophisticated knowledge discovery and data mining (KDD) applications (Holmes, 2014; Sacchi and Holmes, 2016). Exploratory data mining (EDM), a sub‐discipline of knowledge discovery, requires an extensive exploration stage, using both intelligent and intuitive techniques, before predictive modelling and confirmatory analysis can realistically and usefully be applied (De Bie, 2013, 2013; De Bie and Spyropoulou, 2013). Furthermore, it is considered important to include the end user into the ‘interactive’ knowledge discovery process with the goal of supporting human intelligence with artificial intelligence (Holzinger and Jurisica, 2014). Several reports have described the benefits attained by leveraging the unique human cognitive capabilities we have, both within the fields of pattern recognition and higher‐order reasoning, to interpret complex biological data and help extract biologically meaningful interpretations (Isenberg *et al.,* 2013; Lee *et al.,* 2012). Visualizing biological information in a concise format and user‐centred design can help achieve this (Fox and Hendler, 2011; Pavelin *et al.,* 2012).

There are, however, a few important research challenges that need resolving before KDD and EDM techniques can optimally be applied to KGs. These include the formalization of concepts such as an ‘interesting pattern’ found in a genome‐scale KG, since ‘interestingness’ is subjective and will depend on the user’s perspective. The concept of ‘explaining a specific biological story’ using a minimum set of non‐redundant and relevant patterns from the KG also needs to be formalized. These theoretical insights need to be turned into useful, scalable and interactive tools, suitable for use by non‐experts and tested against real biological problems.

**Figure 1:**
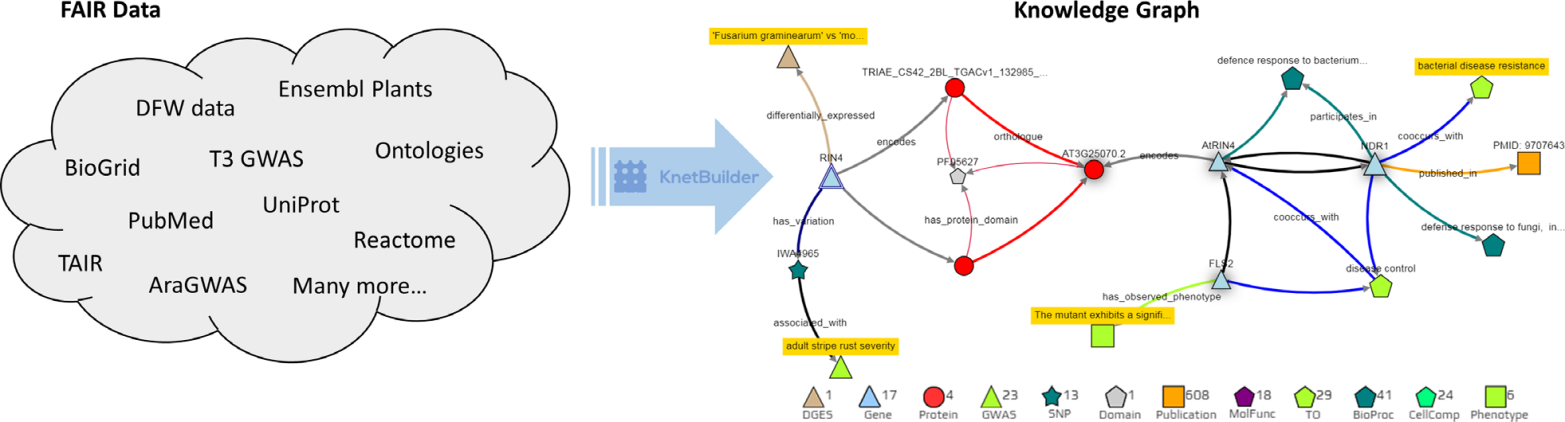
Diverse and heterogenous FAIR data sets are harmonized into a knowledge graph using the KnetBuilder software. Genome‐scale knowledge graphs contain all the genes of an organism with links to functional information across species (Hassani‐Pak *et al.,* 2016). The Illustration shows a single gene knowledge network containing many biological labels and relation types.

We have previously described our approaches (Figure 1) to build genome‐scale KGs with the KnetBuilder (https://github.com/Rothamsted/knetbuilder) data integration platform (Hassani‐Pak *et al.,* 2016), to extend KGs with novel gene–phenotype relations from the literature (Hassani‐Pak *et al.,* 2010), to publish KGs as standardized and interoperable data based on FAIR principles (Brandizi *et al.,* 2018a) and to visualize biological knowledge networks in an interactive web application (Singh *et al.,* 2018). Our data integration approach to build KGs is based on an intelligent data model with just enough semantics to capture complex biological relationships between genes, traits, diseases and many more information types derived from curated or predicted information sources. In this paper, we describe the KnetMiner gene discovery platform (knetminer.org) for searching large genome‐scale KGs and visualizing interesting subgraphs of connected information about the biology of genes, gene networks, traits and diseases. KnetMiner is customizable and portable and therefore provides a cost‐effective delivery platform for application to new species and datasets. We provide use cases to demonstrate how KnetMiner has helped scientists to tell the story of complex traits and diseases in *Arabidopsis thaliana* and *Triticum aestivum* (bread wheat). The methods section describes the algorithms behind core features of KnetMiner, that is, identifying interesting subgraphs and using these to rank candidate genes.

## Results

**Figure 2:**
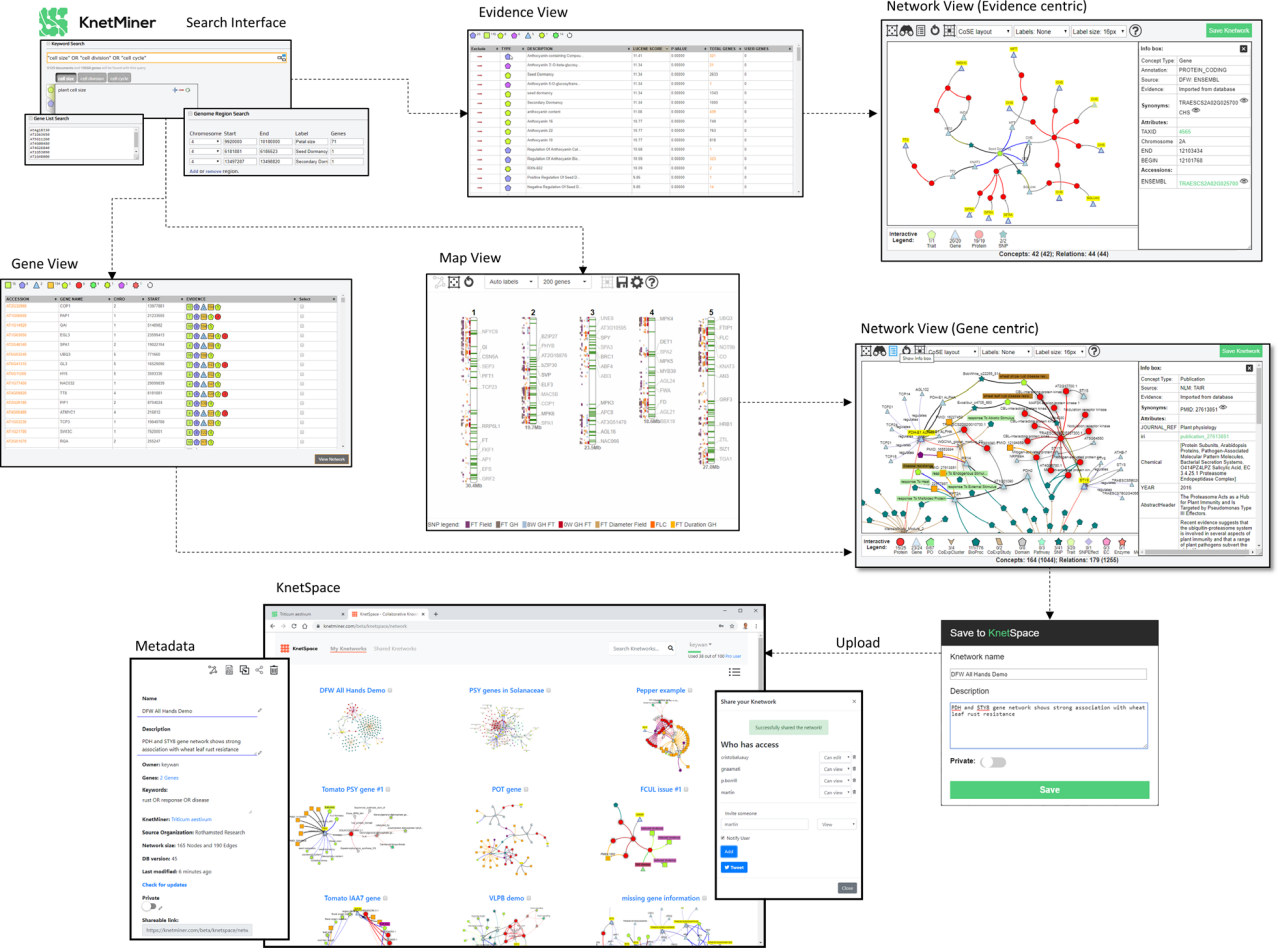
User journeys in KnetMiner. Users start with a search for keywords, genes and regions. KnetMiner provides search term suggestions and real‐time query feedback. From a search, a user is presented with the following views: *Gene View* is a ranked list of candidate genes along with a summary of related evidence types. *Map View* is a chromosome‐based display of QTL, GWAS peaks and genes related to the search terms. *Evidence View* is a ranked list of query‐related evidence terms and enrichment scores along with linked genes. By selecting one or multiple elements in these three views, the user can get to the *Network View* to explore a gene‐centric or evidence‐centric knowledge network related to their query and the subsequent selection. The *Network View* has a set of features for exploring highly connected and rich biological information. For example, the ‘Info Box’ shows the properties of nodes and edges and provides hyperlinks to core databases. The ‘Interactive Legend’ allows users to add or hide information on a whole network level, and, a right‐click radial menu allows to do this for individual nodes and edges. The ‘Save Knetwork’ button allows users to save a gene network, along with metadata and layout information to their workspace (named KnetSpace). In KnetSpace, users can manage all gene networks saved from various KnetMiner resources, edit networks, share with other users and publish online, for example https://knetminer.com/beta/knetspace/network/b21cd8b5‐9eb8‐4713‐aefc‐4785f4c8c8f7.

KnetMiner can assist in various stages of a typical gene discovery project: from the early stages of literature review and hypothesis generation to later stages of biological understanding and hypothesis validation. The user‐centric web interfaces support user journeys for the exploration of complex connected data. An initial simple search interface triggers a sophisticated search process and steps the user from generation to publication of interactive gene networks (Figure 2). We have selected two biological case studies that show the application of KnetMiner in gene–trait discovery and candidate gene prioritization in a model and non‐model species. A detailed description of the latest KnetMiner features is available in the File S1 or online user tutorial.

### Gene network discovery

KnetMiner is being used extensively to drive gene–trait discovery research in the publicly funded ‘Designing Future Wheat’ programme (https://designingfuturewheat.org.uk/), see for example (Adamski *et al.,* 2020; Alabdullah *et al.,* 2019; Harrington *et al.,* 2020). Wheat (*Triticum aestivum*) is the third most‐grown cereal crop in the world after maize and rice, and has a hexaploid 15 Gb genome which is 5 times the size of the human genome (The International Wheat Genome Sequencing Consortium (IWGSC) *et al.,* 2018). As an example, white‐grained wheat varieties lack the red compounds (flavonoids) of the seed coat and are milder in flavour. However, white grains are prone to pre‐harvest sprouting (PHS) which causes the grain to germinate before harvest and results in a loss of grain quality. It has been known for some time that PHS is associated with grain colour (Nilsson‐Ehle, 1914), and that the red pigmentation of wheat grain is controlled by *R* genes on the long arms of chromosomes 3A, 3B and 3D (Sears, 1944). However, after decades of research, it remains unclear whether there is a potential link between the grain colour gene R (Myb) and other phenotypes such as PHS.

We used KnetMiner to search for TRAESCS3D02G468400 (https://knetminer.org/wheatknet/genepage?list=TRAESCS3D02G468400) – the wheat *R* gene (the orthologue of Arabidopsis *TT2*) on chromosome 3D, and to explore its knowledge network as generated by KnetMiner. Our generated *TT2* network has a total of 823 connected nodes of 11 different types (see Table S1) including wheat‐specific information sources but also cross‐species information from model organisms such as Arabidopsis and rice. Furthermore, a range of relation types are present in the network including homologies, transcription‐factor target relations, protein–protein interactions, phenotypic observations and correlations from mutant and genetic studies, as well as, curated or auto‐generated links to ontology terms and publications.

**Figure 3:**
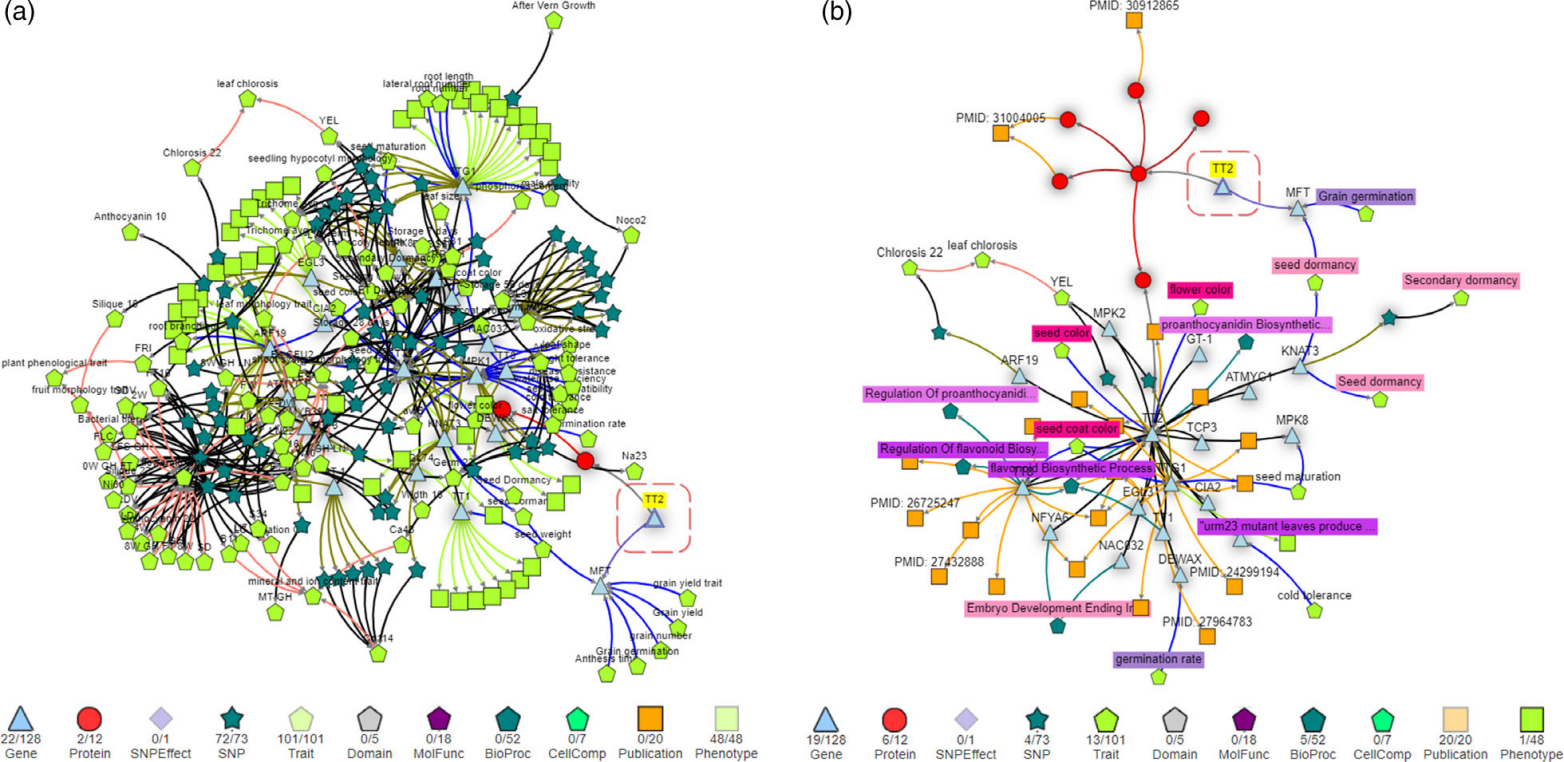
KnetMiner has two modes for generating gene networks depending on whether keywords are provided. (a) TT2 gene network without additional keywords shows all paths to traits and phenotypes. (b) TT2 gene network with keywords relevant to PHS and grain colour traits shows a smaller and more specific network including publications, traits, phenotypes, GO terms, pathways and more.

To reach the final network visualization, KnetMiner first searches for interesting subgraphs generated from the user‐provided genes and keywords (see Methods—Graph Interestingness). In this example, the *TT2* gene search was performed without additional keywords, KnetMiner therefore only shows paths in the gene network that lead to traits and phenotypes. This trims the network from 823 nodes down to 245 nodes including 101 Traits, 48 Phenotypes, 72 SNPs, 22 Genes and 2 Protein nodes (Figure 3a). This network is ultimately displayed in the Network View which provides interactive features to hide or add specific evidence types from the network. Graphical nodes are displayed in a meaningful combination of shapes, colours and sizes to distinguish different types of evidence. A shadow effect on nodes indicates that more information is available but has been hidden. However, the auto‐generated network is not yet telling a story that is specific to our traits of interest and it is limited to evidence that is phenotypic in nature.

To highlight extra ways to use Knetminer and to further refine and extend the search for evidence that links *TT2* to grain colour and PHS, we can provide additional keywords relevant to the traits of interest. Seed germination and dormancy are the underlying developmental processes that activate or prevent pre‐harvest sprouting in many grains and other seeds. The colour of the grain is known to be determined through accumulation of proanthocyanidin, an intermediate in the flavonoid pathway, found in the seed coat. These terms and phrases can be combined using Boolean operators (AND, OR, NOT) and used in conjunction with a list of genes. Thus, we search for TRAESCS3D02G468400 (https://knetminer.org/wheatknet/genepage?list=TRAESCS3D02G468400&keyword=dormancy%20germination%20color%20flavonoid%20proanthocyanidin) and the keywords: *‘seed germination’ OR ‘seed dormancy’ OR colour OR flavonoid OR proanthocyanidin*. This time, KnetMiner filters the extracted *TT2* gene network (823 nodes) down to a smaller subgraph of 68 nodes and 87 relations in which every path from *TT2* to another node corresponds to a line of evidence to phenotype or molecular characteristics based on our keywords of interest (Figure 3b).

This auto‐generated subgraph visualizes complex information in a concise and connected format, helping facilitate biologically meaningful conclusions between *TT2* and phenotypes such PHS (see Table S2). The subgraph indicates that *TT2* in wheat is predicted to regulate the transcriptional activation of *MFT*. If you click on the ‘cooccurs_with’ edge between *MFT* and the linked trait nodes, you can see in the ‘Info Box’ that *MFT* has been linked in a recent publication to grain germination and seed dormancy in wheat (Li *et al.,* 2014; Nakamura *et al.,* 2011). The evidence sentences and hyperlinks to publications can be accessed by clicking links provided in the Info Box. The graph also reveals that the *MFT* ortholog in Arabidopsis is linked to decreased germination rate in the presence of ABA (Xi *et al.,* 2010) and positive regulation of seed germination. To investigate potential links between grain colour and other phenotypes, the TT2 gene network can be expanded with two clicks using the Interactive Legend (see User Tutorial), to add interacting genes in wheat or model species along with their phenotypic information. For example, the Arabidopsis *TT2* ortholog is shown to interact with *TTG1* which has links to phenotypes such as lateral root number and root hair length in Arabidopsis (Bahmani *et al.,* 2016; Bipei Zhang, 2017). Root hairs are tubular outgrowths from specific epidermal cells that function in nutrient and water absorption (Larry Peterson and Farquhar, 1996).

Overall, the exploratory link analysis has generated a potential linkage between grain colour and PHS due to *TT2‐MFT* gene interaction and suggested a new hypothesis between two traits (PHS and root hair density) that were not part of the initial investigation and previously thought to be unrelated. Furthermore, it raises the possibility that *TT2* mutants might have more root hairs and higher nutrient and water absorption, and therefore cause early germination of the grain. More data and experiments will be needed to address this hypothesis and close the knowledge gap.

### Candidate gene prioritization

Forward genetics studies, such as a genome‐wide association study (GWAS) or quantitative trait loci (QTL) mapping, aim to identify regions in the genome where the genetic variation correlates with variation observed in a quantitative trait (e.g. general intelligence, days to flowering) (Atwell *et al.,* 2010; Polderman *et al.,* 2015; Sonah *et al.,* 2015). They are based purely on statistical tests and do not use biological understanding in considering candidates. It is often difficult to elucidate which exact marker is actually biologically significant, particularly in the face of epistatic and epigenetic effects which are often not considered. GWAS and QTL regions can encompass many seemingly unrelated genes. Candidate gene analysis aims to identify the most likely cause for the phenotypic variation. The identification of candidate genes underlying QTL is not trivial; therefore, genetic studies often stop after QTL mapping or perform a basic search for genes with potentially interesting annotations.

**Figure 4:**
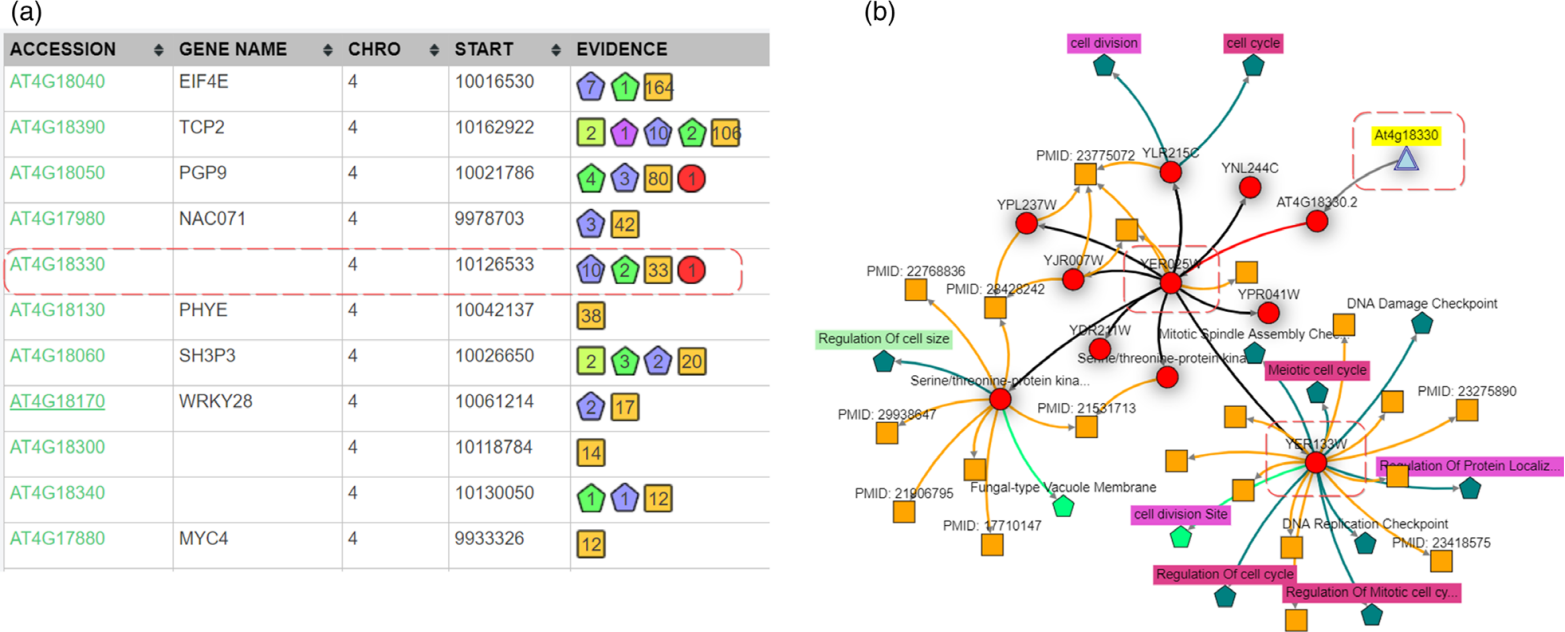
From QTL to candidate genes to gene networks. (a) Candidate genes within a petal size QTL are ranked based on their KnetScore. The Evidence column summarizes the related information found across species. AT4G18330 is linked to 10 biological processes, 2 cellular components, 33 publications and 1 protein related to ‘cell size’ OR ‘cell cycle’ OR ‘cell division’. (b) Interactive gene network for AT4G18330 and ‘cell size’‐related keywords. All publications are linked to the yeast ortholog. The yeast ortholog YER025W interacts with several cell size, cell division and cell cycle‐related proteins.

For example, in a recent QTL study in Arabidopsis, a region on chromosome 4 was identified that contained overlapping QTLs for multiple petal traits (Abraham *et al.,* 2013). As this QTL overlapped with the *ULTRAPETALA1* (*ULT1*) locus, a known floral meristem regulator with a role in petal development (Fletcher, 2001), the authors tested whether *ULT1* might be responsible for this QTL. However, the authors stated that among the ecotypes used in the study none showed any polymorphic sites within the *ULT1* coding or 2kb upstream region; and the T‐DNA insertional mutation of *ULT1* showed no significant effect on petal form either. Taken together, the evidence suggested that *ULT1* was not responsible for the petal size QTL, and the causal gene remained unidentified as is the case in many other GWAS and QTL studies. Therefore, to explore this further, we analysed an overlapping petal size QTL (manuscript in preparation) using a more sophisticated and evidence‐based search to see whether the authors may have missed something. The biological processes underpinning the size of plant tissues and organs are likely to be related to changes on a cellular level. We therefore used, as inputs to KnetMiner, the location of a petal size QTL (chromosome 4, 9.92–10.18 Mb) and the keywords *‘cell size’ OR ‘cell cycle’ OR ‘cell division’*. KnetMiner identified 71 genes in the QTL region and ranked them according to their relevance to the keywords (Figure 4a) (see Methods—Gene Ranking).

The top five highest ranked genes by KnetMiner included a poorly studied gene (AT4G18330) with no links to publications in Arabidopsis and a few high‐level GO annotations. However, the KnetMiner subgraph for AT4G18330 indicated that the yeast ortholog YER025W (eIF‐2‐gamma) interacts with cell division cycle proteins such as CDC123 (Figure 4b). Although no knockouts were available for this gene, a polymorphism in the regulatory region was associated with altered cellular and petal phenotypes consistent with a role in petal size (manuscript in preparation). The ability to both systematically and visually evaluate different layers of evidence arising from orthologs to interactions is highly advantageous; it is quick to view, and as such, the most relevant genes can immediately be investigated further.

## Methods

### Graph pattern mining

We have previously described our tools and methods to build FAIR genome‐scale Knowledge Graphs (KG) using the KnetBuilder and rdf2neo data integration platforms (Brandizi *et al.,* 2018a, 2018b; Hassani‐Pak *et al.,* 2016). Here, we elaborate how KnetMiner uses the KG to extract biologically meaningful subgraphs that tell the story of complex traits and diseases. Biologically, plausible patterns in the KG are collections of paths through the connected information that most biologists would generally agree to be informative when studying the function of a gene. Searching a KG for such patterns is akin to searching for relevant sentences containing evidence that supports a particular point of view within a book. Such evidence paths can be short; for example, Gene A was knocked out and phenotype *X* was observed; or alternatively, the evidence path can be longer; for example, Gene A in species *X* has an ortholog in species *Y*, which was shown to regulate the expression of a disease‐related gene (with a link to the paper). In the first example, the relationship between gene and disease is directly evident and experimentally proven, while in the second example, the relationship is indirect and less certain but still biologically meaningful. There are many evidence types that should be considered for evaluating the relevance of a gene to a trait. In a KG context, a gene is considered to be, for example, related to ‘early flowering’ if any of its biologically plausible graph patterns contain nodes related to ‘early flowering’. In this context, the word ‘related’ does not necessarily mean that the gene in question will have an effect on ‘flowering time’, but it means that there is a valid piece of evidence that a domain expert should consider when judging whether the gene is related to ‘flowering time’.

We use the notion of a semantic motif to define a plausible path through the KG. Our semantic motifs start with a gene node and end with other nodes representing biological entities, ontology terms, publications etc. For example, a path that travels from a Gene node to a GO‐term, through an ortholog relation, is biologically plausible (orthologs have often the same function), while travelling through a paralog relation is not (paralogs often adapt new functions). KnetMiner instances can have a bespoke set of semantic motifs reflecting the data model of the KG built for one particular species or domain of interest. We are working towards migrating KnetMiner to support the Cypher graph query language and the Neo4j graph database as a practical and expressive way to define the graph searches that capture the semantic motifs of interest. Table S3 contains example Cypher queries used in the public wheat KnetMiner along with summary statistics for each query. The KnetMiner gene search and subgraph generation are essentially based on these well‐defined graph queries. Not every gene will necessarily match all semantic motifs; however, the ones it contains are extracted and their union is taken to produce a gene‐centric subgraph (GCS). For example, the wheat KG has over 114 000 GCSs (one for each wheat gene) with sizes of min = 1, max = 6220 and mean = 181 nodes.

Nodes that are included in a GCS are presumed to be transferable to the gene of interest; in contrast, concepts that are excluded from a GCS (although still part of the KG) are presumed to be irrelevant to the gene in question. Notably, if a semantic motif fails to capture an important biological motif, then downstream knowledge mining applications would not be able to exploit this information.

### Graph interestingness

Even a single GCS with hundreds of nodes can be complex and challenging to comprehend when shown to a user, let alone if combining GCSs for tens to hundreds of genes. There is therefore a need to filter and visualize the subset of information in the GCSs that is most interesting to a specific user. However, the interestingness of information is subjective and will depend on the biological question or the hypothesis that needs to be tested. A scientist with an interest in disease biology is likely to be interested in links to publications, pathways and annotations related to diseases, while someone studying the biological process of grain filling is likely more interested in links to physiological or anatomical traits. To reduce information overload and visualize the most interesting pieces of information, we have devised two strategies. (1) In the case of a combined gene and keyword search, we use the keywords as a filter to show only paths in the GCS that connect genes with keyword‐related nodes, that is nodes that contain the given keywords in one of their node properties. In the special case where too many publications remain even after keyword filtering, we select the most recent N publications (default *N* = 50). Nodes not matching the keyword are hidden but not removed from the GCS. (2) In the case of a simple gene query (without additional keywords), we initially show all paths between the gene and nodes of type phenotype/trait, that is any semantic motif that ends with a trait/phenotype, as this is considered the most important relationship to many KnetMiner users.

### Gene ranking

We have developed a simple and fast algorithm to rank genes and their GCS for their importance. We give every node in the KG a weight composed of three components, referred to as SDR, standing for the Specificity to the gene, Distance to the gene and Relevance to the search terms. Specificity reflects how specific a node is to a gene in question. For example, a publication that is cited (linked) by hundreds of genes receives a smaller weight than a publication which is linked to one or two genes only. We define the specificity of a node *x* as: $S\left(x\right)=\log\;\frac{N}{n}$ where *n* is the frequency of the node occurring in all N GCS. Distance assumes information which is associated more closely to a gene can generally be considered more certain, versus one that is further away, for example, inferred through homology and other interactions increases the uncertainty of annotation propagation. A short semantic motif is therefore given a stronger weight, whereas a long motif receives a weaker weight. Thus, we define the second weight as the inverse shortest path distance of a gene g and a node *x*: $D\;\left(g,x\right)\;=\;\frac{1}{\left|v_{g}\;\to \;v_{x}\right|}$. Both weights *S* and *D* are not influenced by the search terms and can therefore be pre‐computed for every node in the KG. Relevance reflects the relevance or importance of a node to user‐provided search terms using the well‐established measure of inverse document frequency (IDF) and term frequency (TF) (Salton and Yang, 1973). TF*IDF forms the basis of the Lucene search engine library (https://lucene.apache.org/), used in KnetMiner. We define the relevance of node *x* to a search term *t* as R(t,x) = TF×IDF(t,x), where *R* = 0 when no match is found and *R* = 1 when the user does not provide any keywords. The three measures (*S*, *D* and *R*) have unique and uncorrelated characteristics. Each node in KnetMiner is given a combined SDR weight. Therefore, for a given GCS $X_{g}=\left\{x_{1,}x_{2},...,x_{n}\right\}$ and search terms *t*, we define the *KnetScore* of a gene as:

$$KnetScore\left(t,X_{g}\right)=\sum_{x_{i}∊X_{g}\cap x_{i}∋t}S\left(x_{i}\right)*D\left(g,x\right)*R\left(t,x_{i}\right)$$

The sum considers only GCS nodes that contain the search terms. In the absence of search terms, we sum over all nodes of the GCS with *R* = 1 for each node. The computation of the KnetScore (*SDR* weights) requires graph traversals and string searches over the KG. Performing these operations on‐the‐fly would slow down the responsiveness of the application. Therefore at initialization, KnetMiner pre‐processes the KG and builds indices to speed up the *SDR* weight calculation. The pre‐indexing time depends on a number of factors including number of available cores, the KG size, number of genes and number of semantic motifs. With the indices in place, the SDR weight can be computed in constant time O(1). A KnetMiner search that returns n genes and m evidence nodes can rank all genes in linear time O(n + m).

## Discussion

Scientists spend a considerable amount of time searching for new clues and ideas by synthesizing many different sources of information and using their expertise to generate hypotheses. Gene discovery is often hampered by the challenges of data integration, and new approaches are needed to improve the efficiency, reproducibility and objectivity of the process that leads to new ideas and hypotheses. KnetMiner provides a sophisticated search across a semantically rich knowledge graph built from large scale integration of public and private data sets. It addresses the needs of scientists who may lack the time and the broad expertise that is necessary to connect, explore and compare the wealth of genetic, ‘omics, and phenotypic information available in the literature and a wide range of related biological databases from key model and non‐model species.

KnetMiner is commonly used by scientists in academia and industry to accelerate gene–trait discovery research. In several biological studies, KnetMiner enabled the identification of hidden relationships between important agronomic traits and potential candidate genes. The presented case studies have shown practical applications of KnetMiner to the understanding of challenging and complex traits in wheat and Arabidopsis. KnetMiner was used in 2014 to investigate traits such as height of biomass willows (Hanley and Karp, 2014) and has more recently become part of a wider roadmap for gene function characterization in crops (Adamski *et al.,* 2020; Harrington *et al.,* 2020). Public KnetMiner resources (e.g. Arabidopsis, wheat and rice) give a flavour of the capabilities that are in KnetMiner. While we have so far mostly concentrated on customizing KnetMiner for plant sciences and crop improvement, the software we have developed is generic and KGs and KnetMiner can readily be built for many species. Compared to biological discovery platforms available for specific species (Carvalho‐Silva *et al.,* 2019; Miller *et al.,* 2017; Mungall *et al.,* 2017), KnetMiner is species‐agnostic and therefore provides a more cost‐effective delivery platform for application to new data sets. For example, we are working on the development of KnetMiner resources for pest insects and fungal pathogens, and, have recently repurposed KnetMiner for mining COVID‐19 biomedical data and literature (Hutson, 2020). KnetMiner is available as a Docker image from DockerHub and can easily be deployed with a provided sample KG.

Different KnetMiner views for exploring the search output have been developed; each view has a different aim and helps address different questions. The main design principle was to divide the visualization into two steps. First, to present the results in formats that are intuitive and familiar to biologists, such as tables and chromosome views, allowing them to explore the data, make choices as to which gene to view, or refine the query if needed. These initial views help users to reach a certain level of confidence with the selection of potential candidate genes. However, they do not tell the biological story that links candidate genes to traits and diseases. In a second step, to enable the stories and their evidence to be investigated in full detail, the Network View visualizes highly complex information in a concise and connected format, helping facilitate biologically meaningful conclusions. Consistent graphical symbols are used for representing evidence types throughout the different views, so that users develop a certain level of familiarity, before being exposed to networks with complex interactions and rich content.

The methods (graph pattern mining, graph interestingness and gene ranking) that power the KnetMiner user interface are also available as API calls and can be used to embed visualizations of gene‐centric subgraphs in third party web applications or to integrate graph analytics and gene ranking in custom workflows. For example, the KnetMiner REST API is used in Ensembl Plants (Bolser *et al.,* 2017), The Triticeae Toolbox (Blake *et al.,* 2016) and GrainGenes (Blake *et al.,* 2019) to link gene sequences to rich gene knowledge graphs. The graph database backend, as well as the FAIR‐based data management policies, is another development in which we are investing our efforts, which have the main advantage of allowing us to build a data asset that has the potential to be useful to a wealth of applications, complementary to KnetMiner. The SPARQL and Cypher endpoints have the benefit of providing a layer of access to data that have a more general use than gene‐centric knowledge exploration and which, for instance, could be obtained with scripts accessing APIs, workflow tools like Galaxy (Afgan *et al.,* 2018) or data analytics workbenches like Jupyter (Kluyver *et al.,* 2016). This is facilitated by adhering to the well‐known good practice of the FAIR principles, which includes the adoption of common data schemas and ontologies (Garcia *et al.,* 2017).

## Conclusion

KnetMiner is an integrated, intelligent, interactive gene and gene network discovery platform, designed to help scientists understand the biological stories of complex traits and diseases across species. The challenges faced in gene discovery for crop improvement are different from work done in drug target discovery in humans. A plethora of diverse crop, insect and pathogen data sets need to be interconnected with well‐curated model species data and the scientific literature information. KnetMiner has been designed to support a diversity of species, data sets and use cases. We see the real value of KnetMiner being in the areas of multi‐dimensional data integration and global optimization where, by comparison, a human cannot hold that much data depth or breadth together and in balance. An expert scientist might read and understand a single journal article better and follow a single thread or conclusion accurately, but it is the integration and balance of the entirety of the indexed knowledge graph where KnetMiner shines brightest.

## Conflicts of interest

The authors declare that they have no competing interests.

## Author contributions

KHP designed the approach as part of his dissertation with CR, collected results and drafted the manuscript. KHP, AS, MB, JH and the KnetMiner team implemented the KnetMiner framework and maintain its public instances. SA and JDP helped to build the Arabidopsis and wheat knowledge graphs. AP and JHD contributed towards the biological use cases. All authors read, reviewed and approved the final manuscript.

## Software availability

Project name: KnetMiner—Knowledge Network Miner.

Project home page: https://knetminer.org

Source code: https://github.com/Rothamsted/knetminer

Docker image: https://hub.docker.com/r/knetminer/knetminer

Deployment instructions: https://github.com/Rothamsted/knetminer/wiki/

Knowledge Graph Endpoints: http://knetminer.org/data

Operating system(s): Platform independent.

Programming language: Java and JavaScript.

Other requirements: Docker.

Licence: MIT.

Any restrictions to use by non‐academics: database licence needed.

## Supporting information

**Table S1** Occurrence of various information types in the wheat TT2 (TRAESCS3D02G468400) gene‐centric subgraph.

**Table S2** Examples of relation types and properties in the keyword‐filtered TT2 (TRAESCS3D02G468400) subgraph.

**Table S3** Example of semantic motifs (in Cypher language) used in KnetMiner with number of matches found in the Wheat Knowledge Graph (Release 45).

**File S1** KnetMiner v4.0 user tutorial.
