# Supplementary material for: KnetMiner: a comprehensive approach for supporting evidence‐based gene discovery and complex trait analysis across species
Source: Plant Biotechnol J. 2021 Apr 5;19(8):1670–8. doi: 10.1111/pbi.13583 (PMC8384599; doi:10.1111/pbi.13583)
Supplement: Supplementary file 1 — Table S1 Occurrence of various information types in the wheat TT2 (TRAESCS3D02G468400) gene‐centric subgraph. [file PBI-19-1670-s003.pdf]

Supplementary Table 1: Information types and instances in the wheat *TT2 (*TRAESCS3D02G468400) gene-centric subgraph.

**Information type**	**Instances**
Publication	452
Gene	128
Trait	101
SNP	73
Biological Process (GO)	52
Phenotype	48
Molecular Function (GO)	18
Protein	12
Cellular Component (GO)	7
Protein Domain	5
SNP Effect	1
