# Supplementary material for: KnetMiner: a comprehensive approach for supporting evidence‐based gene discovery and complex trait analysis across species
Source: Plant Biotechnol J. 2021 Apr 5;19(8):1670–8. doi: 10.1111/pbi.13583 (PMC8384599; doi:10.1111/pbi.13583)
Supplement: Supplementary file 2 — Table S2 Examples of relation types and properties in the keyword‐filtered TT2 (TRAESCS3D02G468400) subgraph. [file PBI-19-1670-s001.pdf]

Supplementary Table 2: Examples of relation types and properties in the keyword-filtered TT2 *(*TRAESCS3D02G468400) subgraph

**Node A**	**Node B**	**Relation Type**	**Relation Properties**
TT2-3D	MFT-3B	regulates	p-value=0.01; evidence:GENIE3; data=850_samples_wheat_rna_seq
TT2-3D	TT2-3B	homoeolog	Ensembl Compara
MFT-3B	Grain germination (CO:0000011)	co-occurs	Recent studies in both Arabidopsis and wheat have uncovered a new role of MOTHER OF FT AND TFL1 (MFT) in seed germination. (PMID: 24932489)
MFT-3B	Seed dormancy (TO:0000253)	co-occurs	Mapping analysis showed that MFT on chromosome 3A (MFT-3A) colocalized with the seed dormancy quantitative trait locus (QTL) QPhs.ocs-3A (PMID :21896881)
AtMFT	Decreased germination rate	has_phenotype	Decreased rate of germination in the presence of ABA. (PMID:20551347)
AtMFT	Positive regulation of seed germination (GO:0010030)	participates_in	Inferred from Biological aspect of Ancestor<br>Inferred from Mutant Phenotype
AtTT2	AtTTG1	interacts	Two-hybrid (PMID: 15255866)
AtTTG1	Lateral root number (TO:0001013)	co-occurs	Moreover, transgenic TTG1-overexpression (TTG1-OX) seedlings exhibited enhanced root length and lateral root number compared to wild-type seedlings grown under normal or stress conditions. (PMID: 23306631)
TT2	TT8 (bHLH)	interacts	Recently the TaMYC1 gene encoding bHLH transcription factor has been isolated from the bread wheat (Triticum aestivum L.) genome and shown to co-locate with the Pp3 gene conferring purple pericarp color. (PMID: 28983311)
