# Supplementary material for: KnetMiner: a comprehensive approach for supporting evidence‐based gene discovery and complex trait analysis across species
Source: Plant Biotechnol J. 2021 Apr 5;19(8):1670–8. doi: 10.1111/pbi.13583 (PMC8384599; doi:10.1111/pbi.13583)
Supplement: Supplementary file 3 — Table S3 Example of semantic motifs (in Cypher language) used in KnetMiner with number of matches found in the Wheat Knowledge Graph (Release 45). [file PBI-19-1670-s004.pdf]

Supplementary Table 3: Example of semantic motifs (in Cypher language) used in KnetMiner with number of matches found in the Wheat Knowledge Graph (Release 45)

<table>
<tr><th>Query</th><th>Paths in KG</th><th>Paths per Gene</th><th>Path Length</th></tr>
<tr><td>(Gene)-[:enc]->(Protein)-[:ortho]-(Protein)<-[:enc]-(Gene)-[:genetic|physical]-(Gene)-[:pub_in]->(Publication)</td><td>9,549,126</td><td>87.6</td><td>11</td></tr>
<tr><td>(Gene)-[:homoeolog|regulates|genetic|physical]-(Gene)-[:has_mutant|has_variation]->(SNP)-[:leads_to]->(SNPEffect)</td><td>3,478,148</td><td>32.4</td><td>7</td></tr>
<tr><td>(Gene)-[:part_of]->(CoExpCluster)-[:enriched_for]->(PlantOntologyTerm)</td><td>2,734,304</td><td>25.1</td><td>5</td></tr>
<tr><td>(Gene)-[:homoeolog|regulates|genetic|physical]-(Gene)</td><td>2,273,339</td><td>21.0</td><td>3</td></tr>
<tr><td>(Gene)-[:homoeolog|regulates|genetic|physical]-(Gene)-[:occ_in]->(Publication)</td><td>2,040,168</td><td>18.7</td><td>5</td></tr>
<tr><td>(Gene)-[:part_of]->(CoExpCluster)-[:enriched_for]->(BioProc)</td><td>1,751,039</td><td>16.2</td><td>5</td></tr>
<tr><td>(Gene)-[:enc]->(Protein)-[:ortho]-(Protein)<-[:enc]-(Gene)-[:genetic|physical]-(Gene)-[:participates_in]->(BioProc)</td><td>1,497,522</td><td>13.7</td><td>11</td></tr>
<tr><td>(Gene)-[:enc]->(Protein)-[:ortho]-(Protein)<-[:enc]-(Gene)-[:genetic|physical]-(Gene)-[:has_variation]->(SNP)-[:associated_with]->(Trait)-[:pub_in]->(Publication)</td><td>1,329,197</td><td>12.2</td><td>15</td></tr>
<tr><td>(Gene)-[:enc]->(Protein)-[:ortho]-(Protein)<-[:enc]-(Gene)-[:genetic|physical]-(Gene)-[:located_in]->(CelComp)</td><td>1,133,500</td><td>10.5</td><td>11</td></tr>
<tr><td>(Gene)-[:enc]->(Protein)-[:ortho]-(Protein)<-[:enc]-(Gene)-[:genetic|physical]-(Gene)-[:has_variation]->(SNP)-[:associated_with]->(Trait)-[:is_part_of]-(Trait)</td><td>1,062,833</td><td>9.8</td><td>15</td></tr>
<tr><td>(Gene)-[:enc]->(Protein)-[:ortho]-(Protein)<-[:enc]-(Gene)-[:genetic|physical]-(Gene)-[:has_function]->(MolFunc)</td><td>1,051,504</td><td>9.7</td><td>11</td></tr>
<tr><td>(Gene)-[:enc]->(Protein)-[:ortho]-(Protein)<-[:enc]-(Gene)-[:pub_in]->(Publication)</td><td>994,535</td><td>9.1</td><td>9</td></tr>
</table>

<table>
<tr><td>(Gene)-[:enc]->(Protein)-[:h_s_s|ortho|xref*0..1]-(Protein)-[:pub_in]->(Publication)</td><td>859,379</td><td>7.9</td><td>7</td></tr>
<tr><td>(Gene)-[:enc]->(Protein)-[:h_s_s|ortho|xref*0..1]-(Protein)-[:has_function]->(MolFunc)</td><td>738,183</td><td>6.8</td><td>7</td></tr>
<tr><td>(Gene)-[:enc]->(Protein)-[:h_s_s|ortho|xref*0..1]-(Protein)-[[:participates_in]->(BioProc)</td><td>589,120</td><td>5.4</td><td>7</td></tr>
<tr><td>(Gene)-[:enc]->(Protein)-[:h_s_s|ortho|xref*0..1]-(Protein)-[:has_domain]->(ProtDomain)</td><td>581,162</td><td>5.3</td><td>7</td></tr>
<tr><td>(Gene)-[:enc]->(Protein)-[:h_s_s|ortho|xref*0..1]-(Protein)</td><td>557,435</td><td>5.1</td><td>5</td></tr>
<tr><td>(Gene)-[:enc]->(Protein)-[:h_s_s|ortho|xref*0..1]-(Protein)-[:located_in]->(CelComp)</td><td>488,173</td><td>4.5</td><td>7</td></tr>
<tr><td>(Gene)-[:enc]->(Protein)-[:ortho]-(Protein)<-[:enc]-(Gene)-[:genetic|physical]-(Gene)-[:has_observ_pheno]->(Phenotype)</td><td>484,240</td><td>4.4</td><td>11</td></tr>
<tr><td>(Gene)-[:enc]->(Protein)-[:ortho]-(Protein)<-[:enc]-(Gene)-[:genetic|physical]-(Gene)-[:has_variation]->(SNP)-[[:associated_with]->(Trait)</td><td>481,474</td><td>9.3</td><td>13</td></tr>
<tr><td>(Gene)-[:homoeolog|regulates|genetic|physical]-(Gene)-[:part_of]->(Path)</td><td>450,971</td><td>4.1</td><td>5</td></tr>
<tr><td>(Gene)-[:enc]->(Protein)-[:ortho]-(Protein)<-[:enc]-(Gene)-[:genetic|physical]-(Gene)-[:cooc_wi]-(Trait)</td><td>429,313</td><td>4.0</td><td>11</td></tr>
<tr><td>(Gene)-[:enc]->(Protein)-[:ortho]-(Protein)<-[:enc]-(Gene)-[:genetic|physical]-(Gene)</td><td>390,646</td><td>3.6</td><td>9</td></tr>
<tr><td>(Gene)-[:enc]->(Protein)-[:ortho]-(Protein)<-[:enc]-(Gene)-[:has_variation]->(SNP)-[[:associated_with]->(Trait)</td><td>334,404</td><td>3.1</td><td>11</td></tr>
<tr><td>(Gene)-[:enc]->(Protein)-[:h_s_s|ortho|xref*0..1]-(Protein)-[:cat_c]->(EC)</td><td>235,234</td><td>2.2</td><td>7</td></tr>
<tr><td>(Gene)-[:enc]->(Protein)-[:ortho]-(Protein)<-[:enc]-(Gene)-[:has_variation]->(SNP)-[:associated_with]->(Trait)-[:pub_in]->(Publication)</td><td>224,482</td><td>2.1</td><td>13</td></tr>
<tr><td>(Gene)-[:homoeolog|regulates|genetic|physical]-(Gene)-[:cooc_wi]-(Trait)</td><td>223,087</td><td>2.1</td><td>5</td></tr>
<tr><td>(Gene)-[:homoeolog|regulates|genetic|physical]-(Gene)-[:inv_in]->(Reaction)</td><td>222,101</td><td>2.0</td><td>5</td></tr>
</table>

(Gene)-[:enc]->(Protein)-[:ortho]-(Protein)<-[:enc]-(Gene)-[:located_in]->(CelComp)	201,102	1.8	9
(Gene)-[:enc]->(Protein)-[:ortho]-(Protein)<-[:enc]-(Gene)-[:participates_in]->(BioProc)	199,505	1.8	9
(Gene)-[:enc]->(Protein)-[:ortho]-(Protein)<-[:enc]-(Gene)-[:has_variation]->(SNP)-[:associated_with]->(Trait)-[:is_part_of]-(Trait)	176,308	1.6	13
(Gene)-[:enc]->(Protein)-[:ortho]-(Protein)<-[:enc]-(Gene)-[:has_function]->(MolFunc)	174,136	1.6	9
(Gene)-[:has_mutant|has_variation]->(SNP)-[:leads_to]-> (SNPEffect)	137,057	1.3	5
(Gene)-[:enc]->(Protein)	133,346	1.2	3
(Gene)-[:part_of]->(CoExpCluster)-[:part_of]->(CoExpStudy)	103,429	1.0	5
(Gene)-[:enc]->(Protein)-[:h_s_s|ortho|xref*0..1]-(Protein)-[:cat_c]->(EC)-[:equ]-(MolFunc)	102,225	0.9	9
(Gene)-[:occ_in]->(Publication)	94,752	0.9	3
(Gene)-[:enc]->(Protein)-[:ortho]-(Protein)<-[enc_10_8_d:enc]-(gene_8:Gene)	70,891	0.7	7
(Gene)-[:enc]->(Protein)-[:h_s_s|ortho|xref*0..1]-(Protein)-[:is_a]->(Enzyme)	54,192	0.5	7
(Gene)-[:homoeolog|regulates|genetic|physical]-(Gene)-[:has_mutant|has_variation]->(SNP)-[:associated_with]-> (Trait)	53,805	0.5	7
(Gene)-[:enc]->(Protein)-[:h_s_s|ortho|xref*0..1]-(Protein)-[:xref]-(Protein)	48,366	0.4	7
(Gene)-[:enc]->(Protein)-[:ortho]-(Protein)<-[:enc]-(Gene)-[:has_observ_pheno]->(Phenotype)	45,438	0.4	9
(Gene)-[:enc]->(Protein)-[:ortho]-(Protein)<-[:enc]-(Gene)-[:cooc_wi]-(Trait)	40,389	0.4	9
(Gene)-[:part_of]->(Path)	14,213	0.1	3
(Gene)-[:inv_in]->(Reaction)	6,274	0.1	3
(Gene)-[:cooc_wi]-(Trait)	5,976	0.1	3
(Gene)-[:has_mutant|has_variation]->(SNP)-[:associated_with]->(Trait)	1,501	0.0	5
