# Supplementary material for: KnetMiner: a comprehensive approach for supporting evidence‐based gene discovery and complex trait analysis across species
Source: Plant Biotechnol J. 2021 Apr 5;19(8):1670–8. doi: 10.1111/pbi.13583 (PMC8384599; doi:10.1111/pbi.13583)
Supplement: Supplementary file 4 — File S1 KnetMiner v4.0 user tutorial. [file PBI-19-1670-s002.pdf]

KnetMiner V4.0

USER TUTORIAL

About KnetMiner

KnetMiner, with a silent "K" and standing for Knowledge Network Miner, is a suite of open-source software tools for integrating and visualising large biological datasets. The software mines the myriad of databases that describe an organism's biology to show links between relevant pieces of information, such as genes, biological pathways, phenotypes, and publications. It aims to provide leads for scientists who are investigating the molecular basis for a particular trait.

Knowledge networks/graphs provide a perfect data structure for heterogeneous, complex, and interconnected biological information. They can be created using the open-source Ondex data integration platform. A knowledge network consists of labelled nodes, such as a gene, pathway, trait, or publication, that are connected through labelled edges, such as encodes, interacts, published-in. Visit our wiki and read Hassani-Pak et al. (2016) and Hassani-Pak et al. (2020) to learn more about how we build knowledge networks. KnetMiner has been used to represent knowledge-graphs for various species, including *Triticum aestivum*, *Arabidopsis thaliana*, *Oryza sativa, F.graminearum, Z.tritici,* and *SARS-CoV-2 & homo-sapiens*.

KnetMiner performs over 70 graph queries of varying depths to find direct or indirect links between genes and user provided search terms. KnetMiner is fast as it uses a graph database and a graph preindexing methodology. KnetMiner is available at https://knetminer.org/.

KnetMiner search interface

The search field of KnetMiner allows users to input any terms related to traits of interest. The terms can be high level descriptions of a phenotypic trait (e.g. heat tolerance) or more specific terms such as biological processes and protein families (e.g. defence response to fungi or LRR). Search terms can be combined with '**OR**', '**AND**', '**NOT**' statements or put into double quotations for exact searches, i.e. *"pathogen" AND "disease"*

Additionally, as the user types a query, the number of resulting documents and genes related to the query are shown and constantly updated in real time. This is only active once the query term is greater than 3 characters in length, updating at each keyboard event. This will 1) help the user to detect spelling mistakes, 2) give a hint if the query term is too general, or too specific prior to the user executing the search, and 3) motivates the user to examine their query and explore different spelling, language, or more complex query statements (AND, OR, NOT, "").

Refining search terms

A hint icon appears at the right end side of the search box to indicate that alternative search terms are available. Click the hint icon to open a tab-based **query suggester**; click it again and it will close. The shown terms are derived from the underlying knowledge network. The query suggester helps users to refine their keywords by suggesting more specific or synonymous terms. For example, using XYZ on the term 'drought' suggests other terms such as 'drought sensitivity' or 'response to water deprivation'. The wizard allows adding, replacing or excluding the new terms. The real-time messaging directly updates when the keywords change to indicate if the new terms would lead to a different number of linked genes.

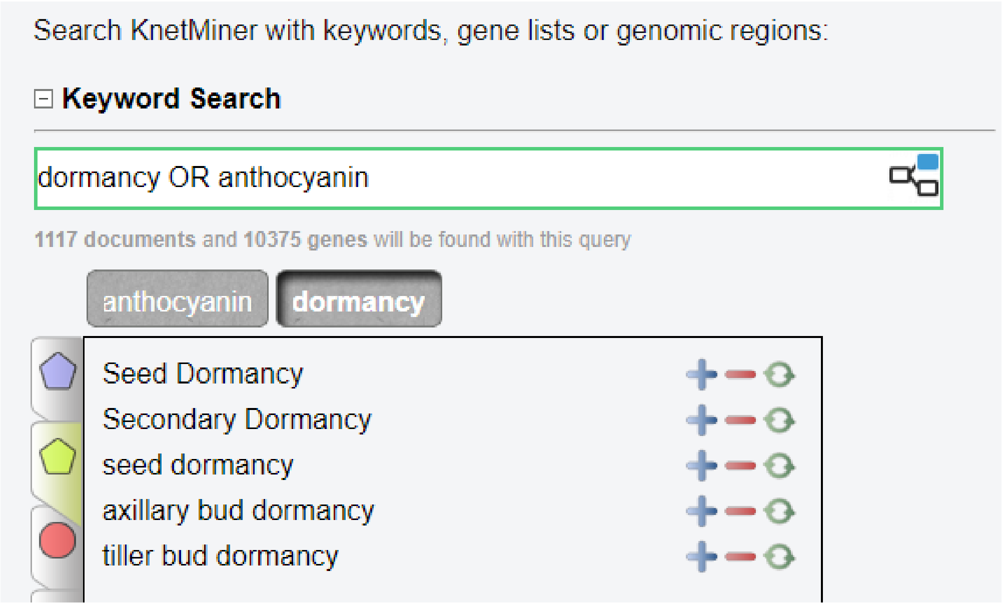

Searching with keyword, gene list and/or genome regions

You can search KnetMiner with keywords, gene list or genome regions (or a**ny** combination of these). KnetMiner will provide different types of responses based on the given inputs. The keyword search will search the whole genome while the other two search modes will be restricted to the specified gene list or genom**ic** regions, respectively.

The gene list search allows users to enter a list of gene names or accessions (limit of 1 entry per line and a maximum of 100 gene ID's). The names/accession IDs need to match (partial matches are **not** enabled – name/accession ID searches are exact) the gene names/ids stored in the knowledge network. Tools like the Ensembl I**D** converter can be used to convert old gene **IDs** to those supported by KnetMiner.

The genome region mode restricts the search to genes that fall within the specified region. Entering the start and end position of a region will display the number of genes within those boundaries. Since **KnetMiner** v3.0, **entering search** keywords **is no longer mandatory**. If you've a list of genes and no clue **about** what they do, just paste your gene ids/names into the Gene List box (without any keyword) and let KnetMiner provide a summary of all information it has for your genes, their location, and enriched linked terms. You can then view their individual knowledge networks. Searching by this method will not rank genes; only paths from gene to trait and phenotype nodes will initially be shown in this instance. However, if you combine your gene list with keywords, KnetMiner will be able to rank your gene list based on relevance and highlight the most interesting paths of the knowledge network.

KnetMiner results views

The result of a search is a list of candidate genes along with supporting evidence. KnetMiner provides different views that help to explore the search results and drill down into interesting candidate gene networks.

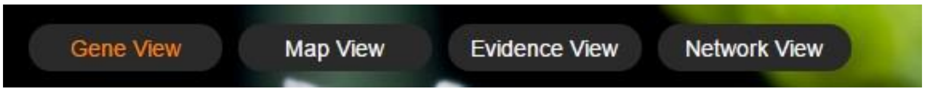

Gene View

The *Gene View* displays identified candidate genes sorted by the KnetScore in a table. The various node types (GO, TO, phenotype, pathway, gene, publication, etc.) matching the search terms are summarised in the legend (below max number of genes dropdown). The legend is interactive and can be used as a filter. Clicking on one or multiple concepts (shapes) in the legend filters the table to genes with matching concepts in the EVIDENCE column, e.g. genes with pathway AND phenotype information. The concepts in the EVIDENCE column are extendible and provide a short description of the evidence. If the evidence is a publication, then the PubMed ID is shown and linked to PubMed.

Genes supplied by a user that are associated with the search terms are referred to as known targets, whereas those user genes that are not associated with any search term (nil evidence) are referred to as novel targets. A checkbox at the top of the Gene View table allows a user to select all known targets or novel targets instantly. Clicking on a single gene, or on the 'View Network' button below, for a selection of genes opens the Network View.

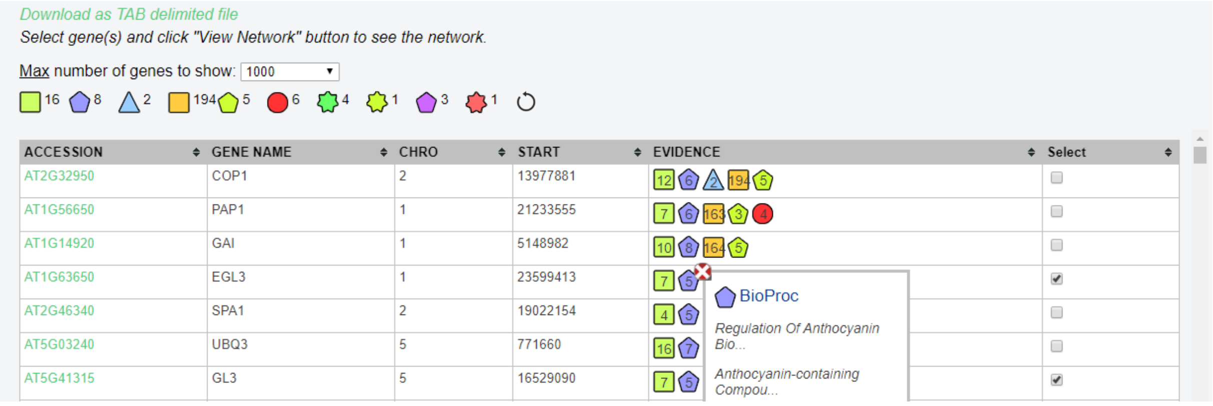

Map View

The *Map View* is the chromosome-based display. To the right of the chromosome, it'll show all the genes which are related to the search term(s) given. Colour coding is used to distinguish genes, with green for high scores, orange for medium, and red for low. SNPs are shown to the left as the highlighted squares, colour coded according to the study shown in the SNP legend, relating to evidence found for the SNP. The user can also specify a specific region search and then only show genes and SNPs within this region. The view can be exported as a PNG, the user can zoom in and out and move across, and the p-value (how significant the association of the gene is with the search term) can be altered via the settings option (cog icon). Right clicking SNPs will provide further information in a pop-up box, where the selected SNP/QTL can be hidden or displayed, as shown below. Genes can be selected in the *Map View* and opened in *Network View* by clicking the network icon on the top (far left).

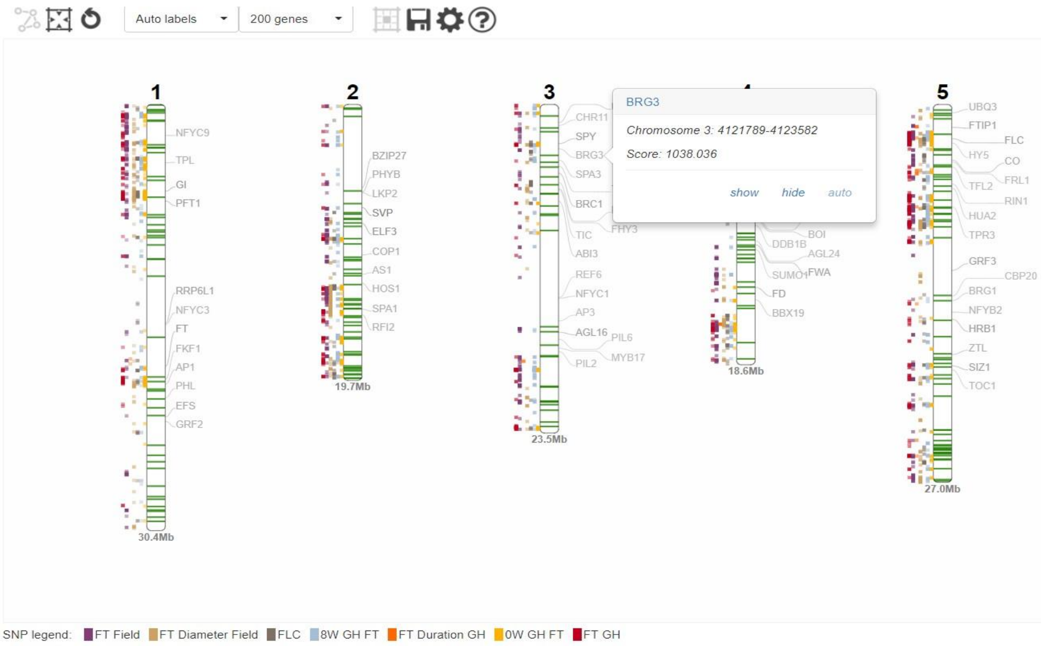

Evidence View

Evidence View provides a table-based view of the node types (concepts) linked to the search results. The results are sorted according to query-relevance score. The number of genes which are linked to each concept in the knowledge network is also displayed (within the 'GENEs' column). Clicking this value will bring you to the *Network View*, containing the selected concepts in the centre of the network with the shortest path which connects the evidence documents to the linked genes. When clicking on a node (concept) icon in the interactive legend, located above the table, the table results will be filtered by evidence type.

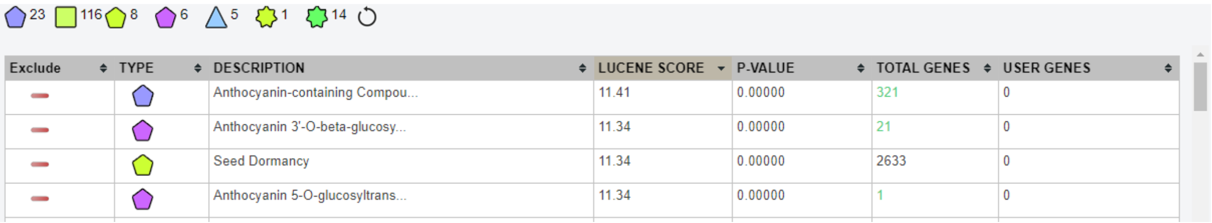

Exclude	TYPE	DESCRIPTION	LUCENE SCORE	P-VALUE	TOTAL GENES	USER GENES
		Anthocyanin-containing Compou...	11.41	0.00000	321	0
		Anthocyanin 3'-O-beta-glucosy...	11.34	0.00000	21	0
		Seed Dormancy	11.34	0.00000	2633	0
		Anthocyanin 5-O-glucosyltrans...	11.34	0.00000	1	0

Network View

The *Network View* will display knowledge networks of one or multiple genes selected from the previous views. The entry gene will be displayed as a blue triangle with a **double border**. Each path starting from the entry gene travelling towards another node will provide a relationship, initially showing only the most relevant relationships to the search term. The maximize button (far left) in the top menu renders the network in a maximised viewport. Should the user click the binoculars, this will show the whole network, but this can cause the application to slow down when loading too many concepts. To view information regarding a concept (node), or its relationship (edge), hold the right click button on the node/edge and a wheel (context menu) will appear. Click 'show info' to see an information table on the right of the network viewport. You can close this by clicking the 'X' button in the info-box. To reset the orientation (zoom) of the graph, click the reset button. The info button next to the 'CoSE layout' drop down menu can also be used to show the info box (you must then click a concept to show information for it).

You can move concepts and edges to be more easily viewable, and you can also save the Knetwork to KnetSpace by pressing the save Knetwork button (it'll prompt you to log in if you aren't already), if

you've signed up. KnetSpace enables you to store, edit, and collaborate on your Knetworks. If you wish to sign in and haven't already logged in via KnetSpace, simply click the top right hand 'Sign in' button on the header.

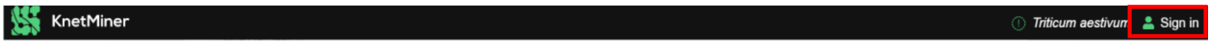

You can also hide concepts or show their labels or hide by their specific type by using the same wheel, or alternatively use the interactive legend where double clicking a concept will remove the concept, and single clicking adds it. On a touchscreen device, gently flick up on the legend concept to remove the concept and tap to add. The concept count will also update to show the current number of concepts present on the graph over the total number, updating as added or removed. The number of concepts and relationships visible are shown below the interactive legend, and the total number are shown adjacent to them in brackets.

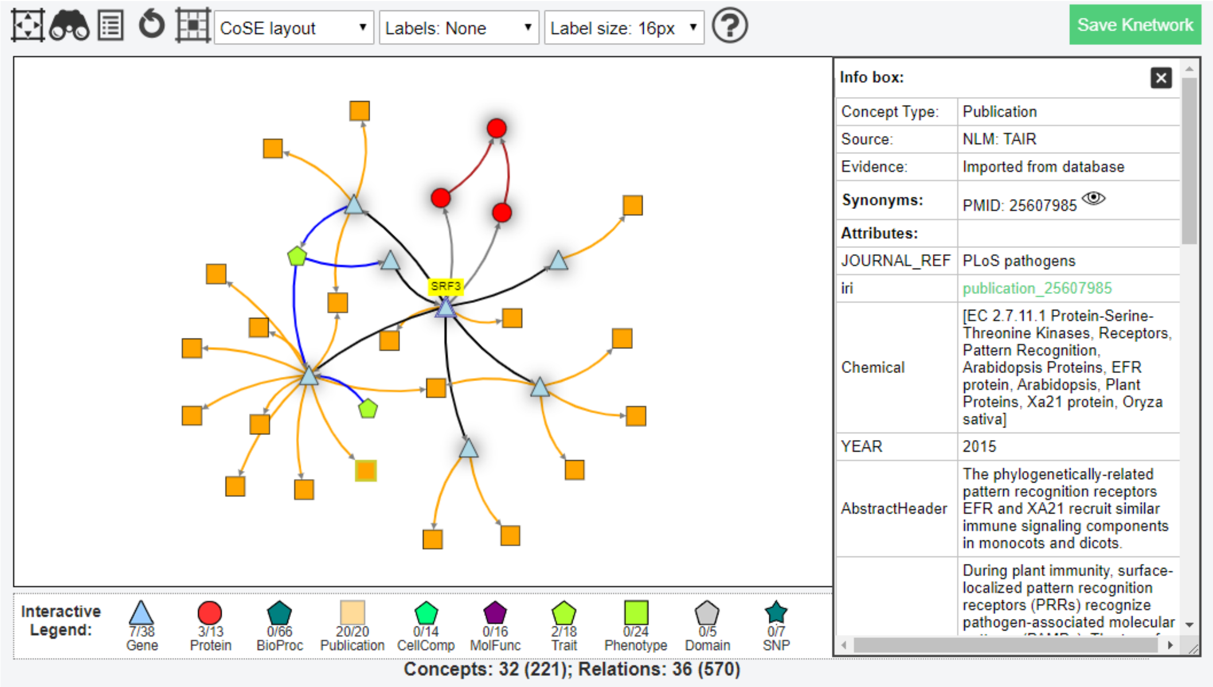

KnetMiner Plant Use Case

This application case shows the utility of KnetMiner for the functional analysis of a transcriptomics (RNA-Seq) experiment in bread wheat (*Triticum aestivum*). Wheat is the third most-grown cereal crop in the world after maize and rice and has a hexaploid genome 5 times the size of the human genome.

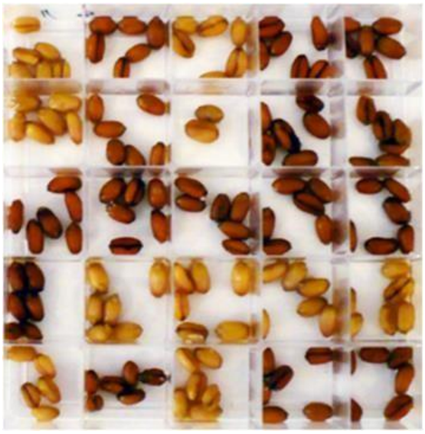

The red colour of the grain is due to the presence of coloured compounds, called flavonoids, in the seed coat (bran). These flavonoids give wholemeal bread not only its colour, but also a slightly bitter taste which is disliked by many people. Whitegrained wheat varieties lack the red compounds of the seed coat and are milder in flavour. However, white grains are prone to pre-harvest sprouting (PHS) which causes the grain to germinate before harvest and results in a loss of grain quality. It has been known for some time that PHS is associated with grain colour

and that the red pigmentation of wheat grain is controlled by R genes on the long arms of chromosomes 3A, 3B, and 3D. In the last decade, the genetic basis of the relationship between grain colour and PHS has been studied and molecular characterisation showed the R gene is a Myb-type transcription factor responsible for transcriptional activation of genes (CHS, CHI, F3H and DFR) in the flavonoid biosynthesis pathway. However, the link between the R (Myb) gene and PHS is still unclear.

Here we demonstrate the utility of KnetMiner for analysing candidate genes from reverse genetics or transcriptomics studies and answering questions such as:

1. Do any of these genes contribute to the expression of trait A (e.g. grain colour)?
2. Do any of these genes contribute to the expression of trait B (e.g. PHS trait)?
3. Which biological processes and pathways are underlying these traits?
4. Are there common genes or mechanisms that influence both traits?
5. Which other processes and traits will be affected by loss-of-function mutants?

Exercise 1 - Choosing the right search terms

Seed dormancy and germination are the underlying developmental processes that activate or prevent pre-harvest sprouting in many grains and other seeds. The user can provide this knowledge as a list of **keywords** into the search box. The **Query Suggester** provides alternative synonyms or more specific keywords. It also highlights key concept types that match the keywords.

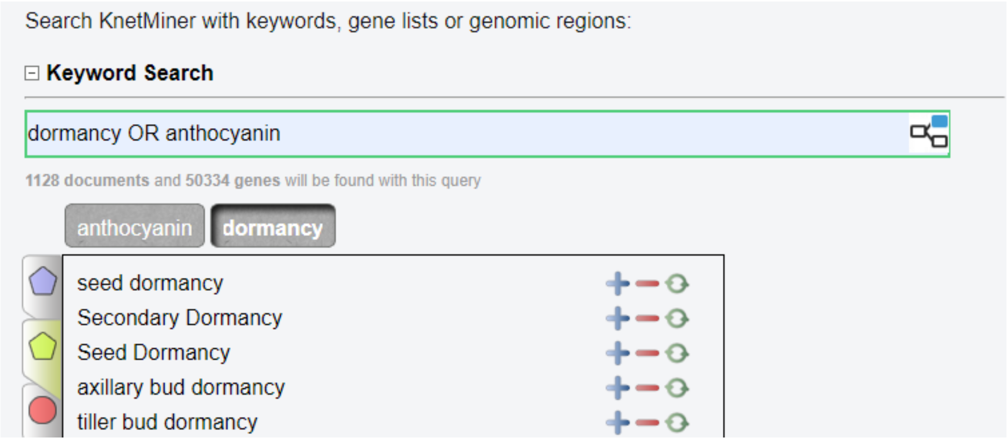

Task: Type the keyword *dormancy* into the search box. Try to replace it with a more specific keyword.

A: The Query Suggester shows that the keyword ***dormancy*** matches Gene Ontology (GO), Trait Ontology (TO), Plant Ontology (PO), gene, and protein concepts from the wheat knowledge network. The TO concepts include terms specific to seed or bud dormancy. It can be assumed that processes involved in grain dormancy are like the ones involved in seed dormancy but different to bud dormancy. Clicking replace changes the keyword to ***"seed dormancy"***, making it more precise.

Exercise 2 – Exploratory analysis of genes supplied by the user

We're now going to explore a list of differentially expressed genes (red *vs.* white grain) in the context of grain colour and PHS traits. The Wheat KnetMiner has the following three example queries that we are going to use:

Example 1 - Grain colour

- color OR flavon* OR proanthocyanidin

Example 2 - Pre-harvest sprouting (PHS)

- "seed germination" OR "seed dormancy"

Example 3 - Grain colour + PHS

- dormancy OR germination OR color OR flavon* OR proanthocyanidin

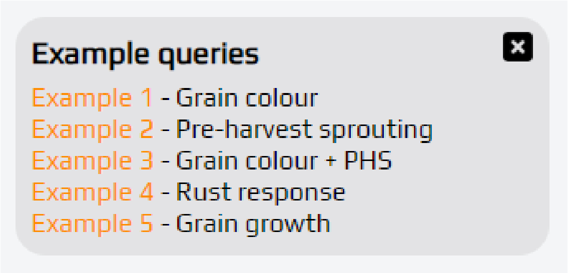

Task: Example 1 populates the search box with search terms and the Gene List with ids. Below the search box you can see two numbers. The first number indicates the nodes in the wheat knowledge network that match the search terms. The second number indicates the number of genes in the wheat genome that have direct or indirect links to these search terms. Press the search button.

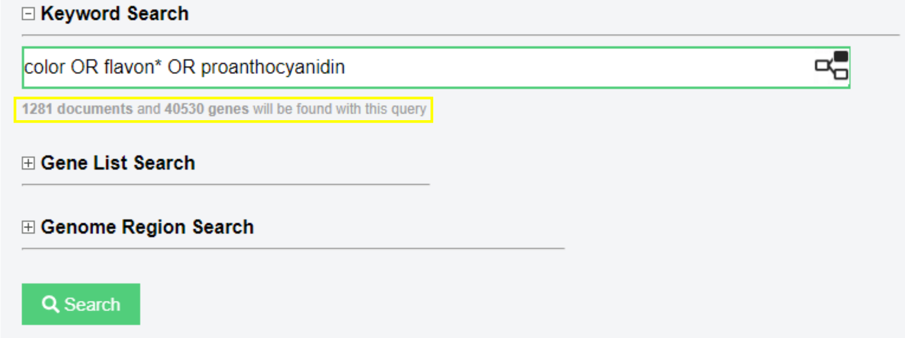

In Gene View, you can use the interactive legend and click on one or more symbols, e.g. pathways, phenotype etc. This will retain genes with the selected evidence types and filter other genes.

→ Which genes are part of the flavonoid biosynthesis pathway?

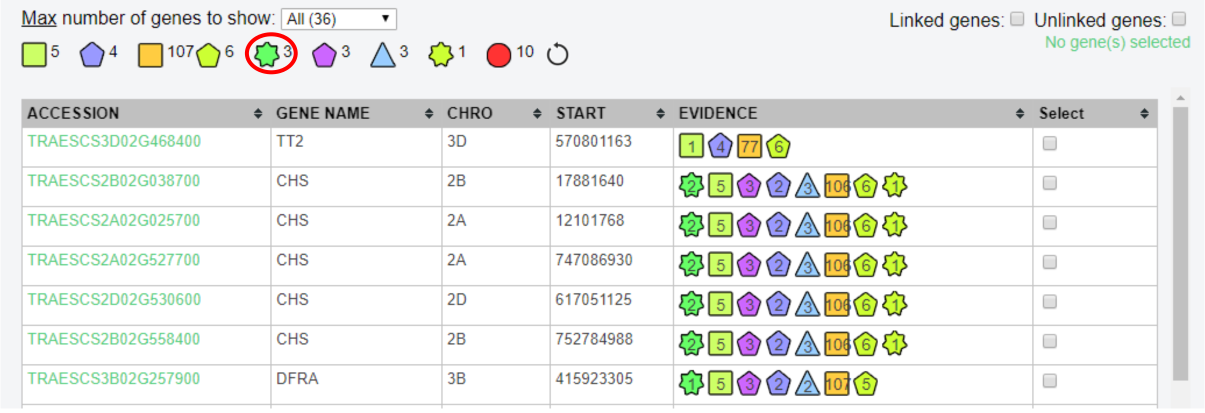

User genes that were not associated with the search terms appear in Gene View with a "0" in the EVIDENCE column.

→ How many user provided genes have known links to Example 1 search terms (known targets) and how many genes have no obvious links (novel targets)?

Repeat these steps for Example 2 – PHS.

Exercise 3 - Exploring gene knowledge networks

We're now going to select single or multiple genes and explore their gene-evidence networks, i.e. the information that links the genes with the search terms.

Task: Click on Example 3 (grain color + PHS) and perform a search. In Gene View, focus on the *TT2* gene (TRAESCS3D01G468400). Check its evidence column and click on it to see the network.

ACCESSION	GENE NAME	CHRO	START	EVIDENCE	Select
TRAESCS3D02G468400	TT2	3D	570801163	1 4 77 6	☑
TRAESCS2B02G038700	CHS	2B	17881640	2 5 3 2 3 106 6 1	☐

→ Can you find the wheat genes (blue triangles with a double border) in the network and follow the path to the Arabidopsis ortholog? Where is the ortholog relation coming from?

→ Which evidence path was used to link the wheat *TT2* gene to pericarp and seed colour?

→ Which evidence path was used to link the wheat *TT2* gene to seed dormancy or grain germination?

→ Which other traits can be affected by *TT2* loss-of-function mutants?

Hint: Enable labels on TO terms that appear as a ***green pentagon***.

A: The knowledge network of wheat *TT2* (R MYB) contains gene regulatory information, protein-protein interactions, phenotypic information in the form of mutant/genetic studies or text-mining, links to relevant ontology terms and publications, and, similar information from Arabidopsis and other species. A more thorough exploration of the information (i.e. node and edge properties) captured in TT2 network tells the following detailed **biological story**:

TT2 (R Myb) on chromosome 3D in wheat is predicted (p-value = 0.01) to regulate the transcriptional activation of MFT according to data from the analysis of 850 RNA-Seq samples in wheat using GENIE3. The TT2 3B homeologue is not predicted to regulate MFT, and the TT2 3A homeologue is not annotated in the latest version of the wheat genome, MFT has been recently linked to grain germination ["Recent studies in both Arabidopsis and wheat have uncovered a new role of MOTHER OF FT AND TFL1 (MFT) in seed germination"] and seed dormancy [Mapping analysis showed that MFT on chromosome 3A (MFT-3A) colocalized with the seed dormancy quantitative trait locus (QTL) QPhs.ocs-3A.]. The MFT ortholog in Arabidopsis has a 3' UTR variant that has been associated with (p-value=5.5x10-5) increased germination rate after 56 days of dry storage.

To discover which other traits will be affected by *TT2* loss-of-function mutants, we can expand the initial *TT2* knowledge graph (click the genes icon in the interactive legend) to add all other genes that are regulated by, or interact with, *TT2*. Other wheat genes regulated by *TT2* don't show any surprising phenotypes. However, the Arabidopsis *TT2* interacts with *TTG1*; a gene known to be involved in controlling root hair density and root hair length in Arabidopsis root hairs. These root hairs are tubular outgrowths from specific epidermal cells, which have important roles in nutrient and water absorption. This interesting clue enables the creation of a speculative hypothesis that pre-harvest sprouting could be caused by increased root hairs, due to higher nutrient and water absorption in *TT2*

knock-outs. This is shown in the gene knowledge network of the *TT2* (R Myb) gene.

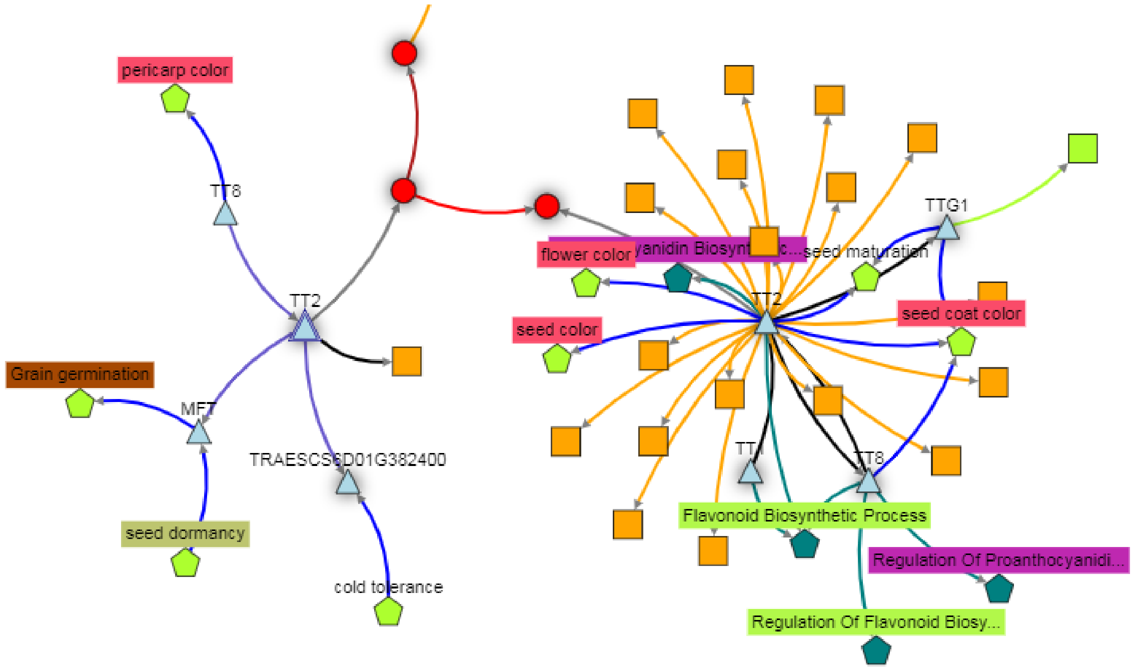

Exercise 4 - Exploring KnetMiner Map View

Task: Click on Example 5 (grain growth) and perform a search. Go to Map View and explore the chromosome, genes, SNP and QTL information displayed for genes ADA2A and BPM5 and then visualize their knowledge network.

1. Click on Example 5 (grain growth) and press Search.
2. Go to Map View and click on gene **ADA2A** on Chr5D and **BPM5** on Chr2D
3. Click on the Network button (top left) and explore the network
4. Add more information using the interactive legend

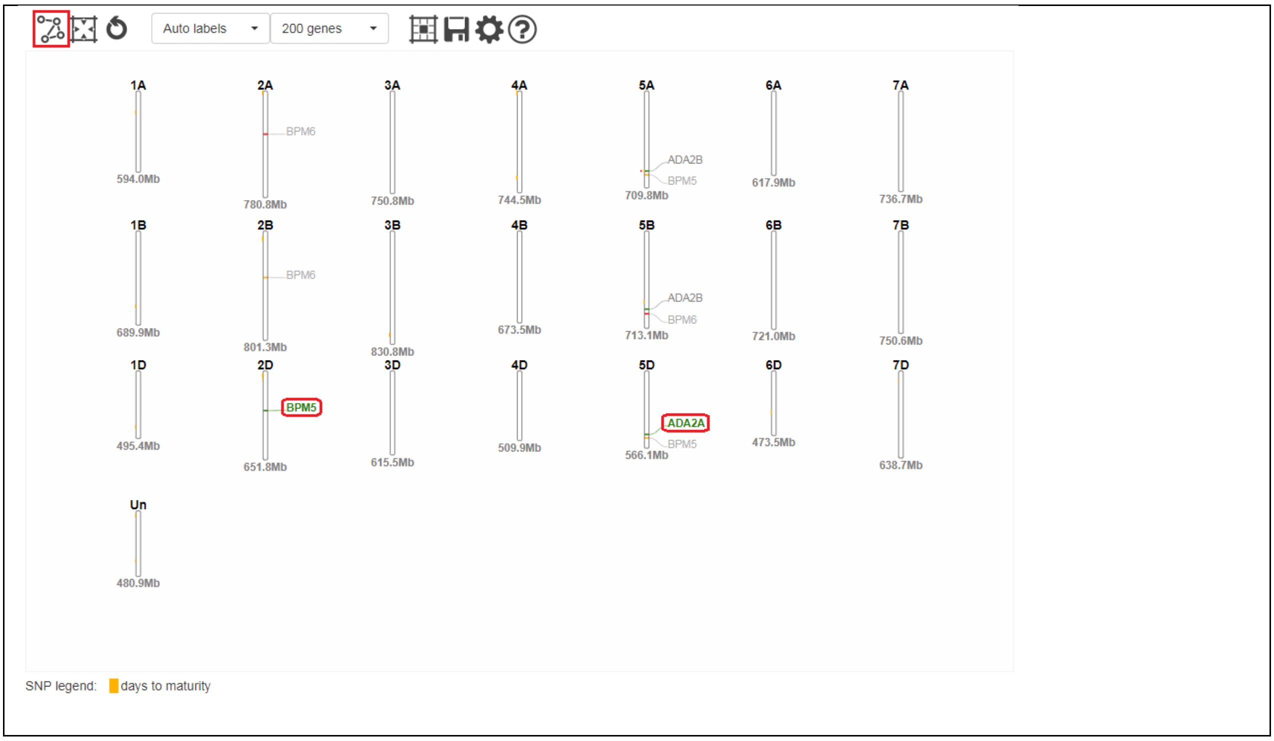
Auto labels
200 genes
1A
594.0Mb
2A
BPM6
780.8Mb
3A
750.8Mb
4A
744.5Mb
5A
ADA2B
BPM5
709.8Mb
6A
617.9Mb
7A
736.7Mb
1B
689.9Mb
2B
BPM6
801.3Mb
3B
830.8Mb
4B
673.5Mb
5B
ADA2B
BPM6
713.1Mb
6B
721.0Mb
7B
750.6Mb
1D
495.4Mb
2D
BPM5
651.8Mb
3D
615.5Mb
4D
509.9Mb
5D
ADA2A
BPM5
566.1Mb
6D
473.5Mb
7D
638.7Mb
Un
480.9Mb
SNP legend: days to maturity
